# Association of temporomandibular disorders and other jaw anomalies in chewing gum users—a systematic review

**DOI:** 10.22514/jofph.2025.022

**Published:** 2025-06-12

**Authors:** Mohammad Khursheed Alam, Maher AL Shayeb, Prabhu Manickam Natarajan, Huda Abutayyem, Marco Di Blasio, Maria Maddalena Marrapodi, Marco Cicciù, Giuseppe Minervini

**Affiliations:** ^1^Preventive Dentistry Department, College of Dentistry, Jouf University, 72345 Sakaka, Saudi Arabia; ^2^Department of Dental Research Cell, Saveetha Institute of Medical and Technical Sciences, Saveetha Dental College and Hospitals, 600077 Chennai, India; ^3^Department of Public Health, Faculty of Allied Health Sciences, Daffodil International University, 1207 Dhaka, Bangladesh; ^4^Department of Clinical Sciences, Center of Medical and Bio-allied Health Sciences and Research, College of Dentistry, Ajman University, P.O. Box 346, Ajman, United Arab Emirates; ^5^Department of Biomedical Surgical and Dental Sciences, University of Milan, 20122 Milan, Italy; ^6^Department of Woman, Child and General and Specialist Surgery, University of Campania “Luigi Vanvitelli”, 80138 Naples, Italy; ^7^Department of Biomedical and Surgical and Biomedical Sciences, Catania University, 95123 Catania, Italy; ^8^Saveetha Dental College and Hospitals, Saveetha Institute of Medical and Technical Sciences (SIMATS), Saveetha University, 600077 Chennai, India; ^9^Multidisciplinary Department of Medical-Surgical and Odontostomatological Specialties, University of Campania “Luigi Vanvitelli”, 80138 Naples, Italy

**Keywords:** Temporomandibular disorders, Gum chewing, Jaw anomalies, Masticatory muscle, Systematic review, Muscle stiffness

## Abstract

**Background**: The relationship between chewing gum and the development of temporomandibular disorders (TMD) and other jaw anomalies presents a contentious topic within dental and orthodontic research communities. This systematic review aimed to synthesize the available evidence regarding the association of gum chewing with the incidence of TMD and jaw anomalies. **Methods**: Adhering to the PRISMA (Preferred Reporting Items for Systematic Reviews and Meta-Analyses) guidelines, we conducted a comprehensive review across six electronic databases—PubMed, EMBASE, Cochrane Library, Web of Science, Scopus and PsycINFO. The studies were chosen based on predetermined inclusion and exclusion criteria, with quality and bias assessments performed on each included investigation. Data extraction and synthesis focused on the relationship between gum chewing habits and the occurrence of TMD symptoms. **Results**: The review included 8 investigations, yielding mixed outcomes. Some studies within this review indicated no direct causative link between the act of gum chewing and the development of TMD-related symptoms, suggesting that symptoms were transient and subsided with the cessation of gum chewing. Conversely, other research suggested a dose-response relationship where increased frequency and duration of gum chewing were associated with escalated TMD symptoms, such as muscle discomfort and hypertrophy. Notably, several studies highlighted the resilience of jaw musculature to adapt to the stress of chewing in individuals without pre-existing TMD, which might be indicative of a protective adaptive response. **Conclusions**: The association between gum chewing and TMD is complex and multifaceted. Evidence from this systematic review suggests a spectrum of effects, from negligible impact to a dose-dependent relationship between gum chewing and TMD symptomatology. **The PROSPERO Registration**: CRD42024553227.

## 1. Introduction

Temporomandibular disorders (TMD) represent a heterogeneous group of musculoskeletal conditions characterized by pain and dysfunction of the jaw muscles, temporomandibular joints (TMJs) and associated structures [1]. These disorders are multifactorial in etiology, encompassing a range of contributing factors including, but not limited to, occlusal discrepancies, psychosocial stress, trauma and parafunctional activities. Chewing gum, as a common masticatory activity, has been postulated to influence the functional dynamics of the masticatory system and has been a subject of scrutiny in the context of TMD and other jaw anomalies [2]. The orofacial complex, an intricate anatomical and functional conglomerate, encompasses a myriad of elements including the osseous structures of the mandible and maxilla, an array of neurovascular bundles, glands associated with saliva production, the musculature responsible for mastication, and the temporomandibular articulations [3]. Among the musculature, there are four principal masticatory muscles: the temporalis, medial pterygoid, lateral pterygoid and masseter muscles. Each muscle originates from cranial structures and converges on the mandibular rami, facilitating the multifaceted actions required for mandibular manipulation [4, 5, 6, 7, 8]. The masseter, notable for its potent contractile capacity, exhibits a quadrilateral configuration and possesses a robust muscular belly, extending from the zygomatic arch to the lateral aspect of the mandible. The temporalis, distinguished by its expansive, fan-like morphology, emanates from the temporal fossa and culminates in a tendinous insertion at the mandibular coronoid process [9, 10, 11, 12, 13].

These muscles are not only integral to the mechanics of mastication but also serve as pivotal components in the broader spectrum of orofacial functions, including phonation and deglutition [14]. Interdisciplinary scrutiny is often necessitated when dysfunctions arise within the orofacial complex, given its functional, structural and anatomical interdependency with contiguous bodily systems. Pathological perturbations within this complex are capable of manifesting distally, implicating extrinsic structures in the ensuing symptomatology [15]. A look at the literature in this regard underscores the ramifications of altered orofacial tension on systemic physiology. It has been further postulated that imprecise proprioceptive feedback originating from the orofacial complex could exert a deleterious influence over cephalic positioning and, by extension, perturb the neural governance of postural stability [16, 17, 18, 19, 20, 21, 22, 23, 24, 25]. The repetitive and often vigorous nature of gum chewing imposes a cyclic load on the TMJs and masticatory muscles, potentially leading to mechanical stress and microtrauma [26, 27, 28]. The impact of this activity on the structural and functional integrity of the masticatory apparatus, however, remains a topic of debate within the scientific community [29]. While some individuals may chew gum without any adverse effects, others may experience exacerbation of pre-existing TMD symptoms or the emergence of new jaw anomalies [28]. Therefore, this systematic review aims to critically appraise and synthesize the current body of literature on the association between gum chewing and the development or exacerbation of TMD and other jaw anomalies. By systematically evaluating evidence from observational and interventional studies, this review additionally endeavors to elucidate the potential pathophysiological mechanisms implied in this relationship and to distinguish between causative, contributory, and incidental associations.

## 2. Materials and methods

### 2.1 Eligibility criteria

This systematic review was conducted following a structured PECO (Population, Exposure, Comparator, Outcome) protocol and adhered to the PRISMA (Preferred Reporting Items for Systematic Reviews and Meta-Analyses) 2020 guidelines, as documented in the **Supplementary material** (PRISMA 2020 Checklist) [30]. This review was registered under the provisional PROSPERO number CRD42024553227.

### 2.2 PECO protocol

• Population (P): The review targeted individuals of all ages who were habitual users of chewing gum.

• Exposure (E): The primary exposure of interest was the habitual act of chewing gum.

• Comparator (C): The comparator group consisted of individuals who did not chew gum or chewed it infrequently, but was not deemed to be mandatory.

• Outcomes (O): The outcomes of interest were the presence of TMD and associated aspects pertaining to TMJ pain, clicking symptoms and discomfort.

### 2.3 Database search protocol

For this review, the database search protocol involved the incorporation of Boolean operators and MeSH (Medical Subject Headings) terms (Table 1). The search strategy was adapted for each database to accommodate the respective syntax and functionalities. The databases searched included PubMed, EMBASE, Cochrane Library, Web of Science, Scopus and PsycINFO. No limitation was placed in terms of the search period of the included articles. Each database search was conducted using a combination of keywords and standardized indexing terms, tailored to the specific database’s interface and indexing system as shown in Table 2.

**Table 1.** Selection criteria devised for this review.

Criteria	Inclusion	Exclusion
Population (P)	Individuals of any age who were habitual users of chewing gum.	Studies focusing on populations with no specified gum chewing habits.
Exposure (E)	Studies examining the habitual act of chewing gum.	Studies without a clear definition of gum chewing habits.
Comparator (C)	Individuals who did not chew gum or who chewed gum infrequently.	-
Outcomes (O)	Presence of TMD and other jaw anomalies (clinical diagnoses or self-reported symptoms).	Studies not assessing TMD or specific jaw anomalies as outcomes.
Study design	Randomized controlled trials, cohort studies, case-control studies, cross-sectional studies.	Editorials, commentaries, reviews, and animal studies.
Language	Studies published in English.	Studies published in languages other than English without a translation.
Publication date	No limitation applied	
Data availability	Studies with available full-text articles and sufficient data for extraction.	Studies with inaccessible full-text or inadequate data for extraction.

*TMD: temporomandibular disorders.*

**Table 2.** Search strings utilised across the different databases under scrutiny for this review.

Database	Search string	Search terms
PubMed	(“Chewing Gum” (MeSH Terms) OR “gum chewing” OR “masticatory activity”) AND (“Temporomandibular Joint Disorders” (MeSH Terms) OR “TMD” OR “temporomandibular disorders” OR “jaw disorders” OR “jaw anomalies”) AND “humans” (MeSH Terms)	MeSH Terms and Boolean Operators
EMBASE	(“chewing gum”/exp OR “gum chewing” OR “mastication”) AND (“temporomandibular joint disorder”/exp OR “TMD” OR “temporomandibular disorder” OR “jaw disorder” OR “jaw anomaly”) AND (humans)/lim	Boolean Operators
Cochrane Library	((“Chewing Gum” (MeSH)) OR “gum chewing” OR “mastication”) AND (“Temporomandibular Joint Disorders” (MeSH) OR “TMD” OR “temporomandibular disorders” OR “jaw disorders” OR “jaw anomalies”)	Title, Abstract, Keywords (ABS) and Boolean Operators
Web of Science	(TI = (chewing gum) OR TI = (gum chewing) OR TI = (mastication)) AND (TI = (temporomandibular joint disorder) OR TI = (TMD) OR TI = (temporomandibular disorder) OR TI = (jaw disorder) OR TI = (jaw anomaly))	Topic Search (TS) and Boolean Operators
Scopus	(TITLE-ABS-KEY (“chewing gum” OR “gum chewing” OR “mastication”) AND TITLE-ABS-KEY (“temporomandibular joint disorder” OR “TMD” OR “temporomandibular disorder” OR “jaw disorder” OR “jaw anomaly”))	Emtree Terms and Boolean Operators
PsycINFO	(“Chewing Gum” OR “gum chewing” OR “mastication”) AND (“Temporomandibular Disorders” OR “TMD” OR “temporomandibular joint disorders” OR “jaw disorders” OR “jaw anomalies”) AND (population (“human”))	Abstract (AB) and Publication Year (PY) and Boolean Operators

*MeSH: Medical Subject Headings; TMD: temporomandibular disorders; TI: Title; KEY: Keywords.*

### 2.4 Variable extraction protocol

A standardised data extraction protocol was devised for this review and tested it on a few studies to make sure it worked well for gathering the necessary information. The aim was to collect a range of information, including study details, methods used, information about the participants, what they were exposed to, how they were compared, what outcomes were measured, what the results were, and what the authors concluded. Two reviewers independently went through the studies and filled out a form with the required information. In cases where the two reviewers did not agree, they talked it over to find a solution or, if needed, they asked for another opinion from a third reviewer.

The Cohen’s kappa statistic was used to check how well the two reviewers agreed on what studies to include and the information they gathered. The results showed that they mostly agreed on which studies to include from the titles and abstracts (κ = 0.85) and which full texts were eligible (κ = 0.80). They had very high agreement when it came to pulling out key information like study design, who was in the study, and the main results (κ = 0.90). These kappa values suggested that the data extraction process was consistent and reliable. The review included these numbers to show the careful approach of the study and to let readers know that the results were not likely to be affected by the reviewers’ personal judgments.

### 2.5 Bias assessment protocol

We implemented a bias assessment protocol to critically evaluate the risk of bias in the included studies which involved the usage of Cochrane Risk of Bias 2.0 (RoB 2.0) tool for randomized control trials (RCTs) [31], while the Risk of Bias in Non-randomized Studies of Exposures (ROBINS-E) tool [32] was applied to non-randomized studies.

### 2.6 Assessment of certainty bias

Upon completing the bias assessment using the Cochrane RoB 2.0 [31] and ROBINS-E [32] tools, the research team proceeded with the Grading of Recommendations Assessment, Development and Evaluation (GRADE) approach [33] to evaluate the overall certainty of the evidence included in this review. The GRADE framework provided a systematic method for considering factors such as study limitations, consistency of effect, imprecision, indirectness and publication bias, which collectively determined the quality of the evidence for each outcome.

### 2.7 Groups and parameters assessed

Al Sayegh *et al*. [34] assessed the impact of chewing tasks of differing durations (40 minutes and 60 minutes) on psychosocial variables, clinical examination of TMD (Diagnostic Criteria (DC)/TMD-Axis I), perceived exertion (RPE), and pain intensity (NRS). The psychosocial variables included assessments for the Generalized Anxiety Disorder-7 (GAD-7), the Patient Health Questionnaire-9 (PHQ-9), the Patient Health Questionnaire-15 (PHQ-15), the Perceived Stress Scale-10 (PSS-10), and the Pain Catastrophizing Scale-13 (PCS-13). This comprehensive approach allowed for a multidimensional analysis of the psychological and physical responses to prolonged chewing tasks. Christensen *et al*. [35] assessed the electromyographic (EMG) activity of the masseter muscles in healthy adults during three functional states: rest (idling), unilateral chewing, and maximum voluntary contraction (MVC). This study provided insights into the muscle activity patterns associated with various functional states of the masseter muscle, which is relevant to understanding masticatory muscle behavior in TMD.

Correia *et al*. [36] differentiated groups based on gum chewing habits and the presence of parafunctional habits. They measured the frequency and duration of gum chewing, the presence of TMD symptoms such as arthralgia and myofascial pain, and the extent of masseter hypertrophy. By comparing these variables across different groups, the study aimed to elucidate the relationship between gum chewing habits and TMD symptomatology. Farella *et al*. [37] examined the effects of chewing tasks (using hard gum, soft gum and empty chewing) on perceived muscle pain and masticatory fatigue. They used the Visual Analog Scale (VAS) to evaluate subjective pain experiences and measured pressure pain thresholds (PPTs) in the masseter and anterior temporalis muscles in women without TMD. This approach helped quantify subjective pain experience and objective muscle sensitivity.

Matsuda *et al*. [38] investigated the EMG activities of the masseter during different gum chewing tasks. The study measured the normalized root mean square (n-RMS) values of rhythmic masticatory muscle activity (RMMA) phasic bursts, along with burst duration and cycle time. This provided a detailed examination of masseter muscle function during mastication. Olchowy *et al*. [39] used shear wave elastography to measure the stiffness of the masseter and temporalis muscles before and after intensive gum chewing, as well as after relaxation. They also assessed the correlation between the stiffness of these two muscles. This study contributed to the understanding of muscle elasticity changes due to masticatory activity.

Watemberg *et al*. [40] examined the daily duration of gum-chewing in different groups and its association with a family history of migraine, findings from neuroimaging studies, and funduscopic examinations. By correlating these variables, the study sought to explore the potential link between gum chewing and neurological conditions. Yashiro *et al*. [41] compared healthy adults and TMD patients in terms of the kinematics of gum-chewing cycles using a non-invasive kinesiograph. They measured positional errors during opening/closing movements, skewness of the velocity profile, and ultraviolet (UV) for these movements. The comparison aimed to identify distinctive kinematic patterns associated with TMD.

## 3. Results

### 3.1 Study selection process

The initial identification of records yielded 417 items from various databases and none from registers (Fig. 1). Before screening commenced, several records were removed: 39 were duplicates, 55 were marked as ineligible by automation tools, and no records were removed for other reasons. This left 323 records to be screened. Upon further scrutiny, 41 records were excluded due to the unavailability of full-texts, which necessitated the retrieval of 282 reports. However, not all reports could be retrieved; 38 remained inaccessible due to restricted access to certain journals and databases that required specific subscriptions or institutional memberships that were not available to us. Consequently, 244 reports were assessed for their eligibility based on the review criteria. During the eligibility assessment, several reports were excluded for specific reasons: 37 did not respond to the PECO criteria set for the study, 46 were off-topic, 65 were individual case reports, 51 were animal studies, and 37 were scoping reviews. After applying these exclusion criteria, only 8 studies [34, 35, 36, 37, 38, 39, 40, 41] met the requirements and were included in the review.

**Fig. 1.** PRISMA flowchart for the review. PECO: Population, Exposure, Comparator, Outcomes.

### 3.2 Bias observed across selected papers

When comparing the two studies, Al Sayegh *et al*. [34] and Correia *et al*. [36], both were evaluated to have an overall low risk of bias, although they each encountered domain-specific concerns (Fig. 2, Ref. [34, 36]). Al Sayegh *et al*. [34] demonstrated a low risk of bias in most domains including the randomization process, adherence to intervention, and outcome measurement. Their concerns were primarily with missing outcome data and the selection of the reported result. Correia *et al*. [36], in contrast, had similar low risks in the randomization process, outcome measurement, and reported results, but their concerns were concentrated on deviations from the intended intervention, which is an aspect where Al Sayegh *et al*. [34] did not face the same level of concern. Despite these individual domain concerns, both studies were ultimately classified as having a low overall risk of bias, suggesting that their findings are relatively robust.

**Fig. 2.** Assessed bias across different domains using the RoB 2.0 tool.

As shown through Fig. 3 (Ref. [35, 37, 38, 39, 40, 41]), comparatively, the studies by Christensen *et al*. [35], Farella *et al*. [37], and Watemberg *et al*. [40] shared similar overall low risks of bias, although each encountered some concerns in different domains. Christensen *et al*. [35] faced issues with the measurement of exposure and missing data, while Farella *et al*. [37] had some concerns regarding bias due to post-exposure interventions. Watemberg *et al*. [40], on the other hand, also had concerns in the domain of post-exposure interventions but were consistent in all other domains. Matsuda *et al*. [38] and Yashiro *et al*. [41] both presented low risks of bias in the majority of domains, with their only concerns being in the measurement of outcomes. These singular concerns did not significantly impact their overall low risk assessments. Olchowy *et al*. [39], however, stood out slightly different with concerns in two key areas: the selection of participants and the selection of the reported result, leading to an overall conclusion of some concerns regarding bias.

**Fig. 3.** Assessed bias across different domains using the ROBINS-E tool.

### 3.3 GRADE assessment

As elucidated through Table 3, for the RCTs, represented by Al Sayegh *et al*. [34] and Correia *et al*. [36], the common finding was that chewing gum might increase TMD symptoms, but the effects appear to be temporary and may not be significant. The risk of bias was rated as “low to moderate” due to some concerns in specific domains, but this did not profoundly affect the overall findings. Inconsistency and indirectness were both deemed low, implying a consistent finding across studies and a direct applicability of the results. Imprecision was also rated as low, indicating that the results are precise enough to be considered reliable. No other factors influenced the certainty domain, which was determined to be low.

**Table 3.** GRADE assessment observations.

Study design	Number of studies	Observed common finding	Risk of Bias	Inconsis-tency	Indirectness	Imprecision	Others	Certainty
RCT	2	Chewing gum may increase TMD symptoms, but effects are temporary and may not be significant	Low to moderate	Low	Low	Low	None	Low
Observ-ational	6	Excessive gum chewing is associated with TMD symptoms, muscle stiffness, and altered muscle function; recovery is generally rapid post-chewing	Low	Low	Moderate	Moderate	None	Low

*TMD: temporomandibular disorders; RCT: randomized control trial.*

The observational studies, including Christensen *et al*. [35], Farella *et al*. [37], Matsuda *et al*. [38], Olchowy *et al*. [39], Watemberg *et al*. [40], and Yashiro *et al*. [41], consistently suggested that excessive gum chewing is linked to TMD symptoms, muscle stiffness, and altered muscle function, though recovery is generally quick after cessation of chewing. The risk of bias for these studies was assessed as low, with a consistent methodology across studies and no significant deviations that would undermine the validity of the findings. However, there was some moderate inconsistency and imprecision, which may be due to the variability in study design, population and outcomes measured. Despite these variances, the overall certainty of the evidence from observational studies was considered low.

### 3.4 Population-associated characteristics

The synthesis of findings from Table 4 (Ref. [34, 35, 36, 37, 38, 39, 40, 41]) reveals the diverse array of research designs and population characteristics, conducted between 1996 [35] and 2021 [22]. RCTs were conducted in Sweden in 2020 [34] and Portugal in 2014 [36], with sample sizes of 31 and 50 participants, respectively, and mean ages of 26 and 23 years respectively. The male to female ratios in these trials were nearly balanced in the Swedish study and heavily skewed towards females in the Portuguese study. Observational studies formed the bulk of the research designs, with studies conducted in the USA in 1996 [35], Italy in 2001 [37], Japan in 2016 [38], and Poland in 2021 [39]. The sample sizes ranged from 8 in the USA study to 50 in the Italy study, indicating a variance in study power and potential impact on the reliability of the findings. The mean ages varied widely, from 27 years in the USA study [35] to 42.1 years in the Japan study [38], which suggests a broad age distribution across studies and potential variability in age-related outcomes. The gender distribution was also varied, from all-female participants in the Italy study [37] to a nearly balanced ratio in the Poland study [39].

**Table 4.** Population characteristics of the included studies in this review.

Study name	Year	Region	Design	Sample size (n)	Mean age (in yr)	Male:Female ratio
Al Sayegh *et al*. [34]	2020	Sweden	RCT	31	26	15:16
Christensen *et al*. [35]	1996	USA	Observational	8	27	3:5
Correia *et al*. [36]	2014	Portugal	RCT	50	23	7:43
Farella *et al*. [37]	2001	Italy	Observational	50	24	All females
Matsuda *et al*. [38]	2016	Japan	Observational	23	42	5:18
Olchowy *et al*. [39]	2021	Poland	Observational	40	40	19:21
Watemberg *et al*. [40]	2014	Israel	Observational	30	12	5:25
Yashiro *et al*. [41]	2005	Japan	Observational	20	26	1:1

*RCT: randomised control trial.*

One cross-sectional study was identified from Israel, conducted in 2014 with a sample size of 30 and a mean age of 12.8 years [40], which is notably younger than the other studies reviewed. The gender ratio here was also skewed towards females. The second study from Japan, conducted in 2005 with a sample size of 20, reported a mean age of 26.6 years and an equal distribution of males and females [41]. This provides a contrast to the earlier Japanese observational study with almost the same sample size but with older participants [38]. The global regions represented include both Western and Eastern societies, as well as European and Middle Eastern countries, allowing for potential cross-cultural comparisons. However, the overall sample sizes are relatively small, with only two studies having 50 participants [36, 37].

### 3.5 Chewing gum effect observed

In the study conducted by Al Sayegh *et al*. [34], the researchers observed a higher incidence of arthralgia following a chewing task, with symptoms decreasing at the 2-hour follow-up mark (Table 5, Ref. [34, 35, 36, 37, 38, 39, 40, 41]). Notably, there was a higher prevalence of myalgia and arthralgia after a 60-minute duration of chewing compared to a 40-minute duration. This suggests that prolonged chewing tasks may exacerbate TMJ symptoms, although there appears to be a recovery or adaptation period post-chewing. Christensen *et al*. [35] reported that the majority of participants experienced weak jaw muscle fatigue during unilateral gum chewing, but they did not report jaw muscle pain. This could imply that while prolonged chewing might induce muscle fatigue, it does not necessarily result in pain, potentially due to the sensitization of muscle nociceptors over time.

**Table 5.** Inferences pertaining to the correlation between chewing gum and TMDs as observed in the included studies.

Study name	Groups Assessed	Parameters Assessed	Effect of Chewing Gum on TMDs Observed	Inference Drawn
Al Sayegh *et al*. [34]	Participants in 40-min and 60-min chewing tasks	- Psychosocial variables (GAD-7, PHQ-9, PHQ-15, PSS-10, PCS-13) - DC/TMD-Axis I clinical examination - Borg’s RPE - NRS for pain intensity	Higher incidence of arthralgia post-chewing task with a decrease in symptoms by the 2-h follow-up. Myalgia and arthralgia were present in higher percentages post 60-min chewing task compared to 40-min.	Excessive chewing may not be a suitable experimental model for pain, as symptoms decreased after the cessation of the task and varied between chewing durations.
Christensen *et al*. [35]	Eight healthy adults	- EMG measurements of masseter muscles during idling, unilateral chewing, and MVC	Weak jaw muscle fatigue experienced by the majority during unilateral gum chewing, but no jaw muscle pains. Sensitization of muscle nociceptors might occur due to prolonged chewing and MVC.	Prolonged unilateral chewing may cause muscle fatigue without pain, questioning the association between gum chewing and TMD-related muscle pain. No support for myofascial pain/dysfunction syndrome found.
Correia *et al*. [36]	- Groups A–E based on gum chewing habits - Group F: Non-gum chewers with other parafunctional habits - Group G: No parafunctional habits	- Frequency and duration of gum chewing - TMD symptoms (arthralgia, myofascial pain) - Masseter hypertrophy	- 63% of Group D reported arthralgia and myofascial pain - 33% of Group C reported arthralgia - 83% of Group A and 27% of Group B reported myofascial pain - All of Group E reported masseter hypertrophy	High frequency and longer duration of gum chewing are associated with increased TMD symptoms and masseter hypertrophy.
Farella *et al*. [37]	Fifteen women without TMD	- Chewing tasks (hard gum, soft gum, empty-chewing) - Perceived muscle pain and masticatory fatigue (VAS) - Pressure pain thresholds (PPTs) of masseter and anterior temporalis muscles	- VAS scores for pain and fatigue increased only during hard gum chewing and returned to baseline after 10 min of recovery - No significant changes in PPTs after any chewing task	Jaw muscles recover quickly from prolonged chewing activity in subjects without TMD, indicating resilience in non-TMD affected populations. Hard gum may cause temporary discomfort.
Matsuda *et al*. [38]	Participants during various gum chewing tasks	- Masseteric EMG activities during different types of gum chewing - n-RMS value of RMMA phasic bursts - Burst duration and cycle time of RMMA	- Smaller n-RMS value of RMMA phasic bursts compared to gum chewing - No significant difference in n-IEMG values between RMMA and gum chewing - Burst duration and cycle time of RMMA significantly longer than gum chewing	RMMA exhibits longer but smaller EMG bursts compared to gum chewing, suggesting a distinctive pattern related to RMMA that differs from gum chewing.
Olchowy *et al*. [39]	Participants undergoing shear wave elastography	- Stiffness measurements of masseter and temporalis muscles at baseline, after intense gum chewing, and after relaxing - Correlation between the stiffness of masseter and temporalis muscles	- Significant increase in muscle stiffness after intense gum chewing, with a significant decrease after relaxation - Stiffness of temporalis muscle significantly lower than that of masseter muscle	Intense gum chewing increases muscle stiffness, which is reversible after relaxation. Shear wave elastography is sensitive in detecting these changes, indicating its potential in assessing masticatory muscle response to stress.
Watemberg *et al*. [40]	- Group 1: Up to 1 h/day - Group 2: 1–3 h/day - Group 3: 3–6 h/day - Group 4: >6 h/day	- Daily duration of gum-chewing - Family history of migraine - Neuroimaging studies - Funduscopic examination	- No significant difference in temporomandibular symptoms among groups based on chewing duration. - Discontinuation of gum-chewing led to complete resolution in 19 out of 30 patients, and some improvement in 7 patients.	The amount of daily gum-chewing did not correlate with headache improvement upon cessation, suggesting other factors may influence TMD-related headaches.
Yashiro *et al*. [41]	- Control Group: 10 healthy adults - Patient Group: 10 TMD patients	- Kinematics of gum-chewing cycles using a non-invasive kinesiograph - Positional errors during opening/closing movements - Skewness of the velocity profile - Unpredictable Variability (UV) for opening/closing movements	Higher average UVs in TMD patients compared to controls during gum-chewing, indicating greater abnormality in chewing movements.	The minimum jerk model could reasonably predict the kinematics of gum-chewing in healthy adults but showed significant errors in TMD patients, suggesting it could be a useful tool in assessing abnormalities in TMD-related movements.

*GAD-7: Generalized Anxiety Disorder-7; PHQ: Patient Health Questionnaire; PSS-10: Perceived Stress Scale-10; PCS-13: Pain Catastrophizing Scale-13; DC/TMD: Diagnostic Criteria for Temporomandibular Disorders; RPE: Rating of Perceived Exertion; NRS: Numeric Rating Scale; EMG: Electromyography; MVC: Maximum Voluntary Contraction; VAS: Visual Analog Scale; n-RMS: Normalized Root Mean Square; RMMA: Rhythmic Masticatory Muscle Activity; IEMG: Integrated Electromyography; UV: ultraviolet.*

Correia *et al*. [36] found that a significant proportion of individuals with frequent gum chewing habits (Groups A–D) reported symptoms of arthralgia and myofascial pain, with the highest reports coming from Group D (63%) for arthralgia and Group A (83%) for myofascial pain. Additionally, all individuals in Group E reported masseter hypertrophy, indicating a potential link between gum chewing habits and the development of musculoskeletal alterations and pain syndromes. In the research by Farella *et al*. [37], it was observed that VAS scores for pain and fatigue increased during the chewing of hard gum but returned to baseline after 10 minutes of recovery. There were no significant changes in PPTs after any of the chewing tasks, which suggests that short-term masticatory exertion does not alter muscle pain sensitivity.

Matsuda *et al*. [38] documented that RMMA during sleep showed smaller n-RMS values of phasic bursts when compared to those during gum chewing. Additionally, the burst duration and cycle time of RMMA were significantly longer than during gum chewing, indicating a distinct pattern of muscle activity in TMD-related muscle actions versus normal chewing. Olchowy *et al*. [39] reported a significant increase in muscle stiffness after intense gum chewing, which significantly decreased after a period of relaxation. Furthermore, the temporalis muscle exhibited significantly lower stiffness compared to the masseter muscle, suggesting that the masticatory muscles respond differently to mechanical stress and recover at different rates.

Watemberg *et al*. [40] found no significant differences in temporomandibular symptoms among the groups based on the duration of gum-chewing. Interestingly, the discontinuation of gum-chewing led to complete resolution of symptoms in 19 out of 30 patients and some improvement in 7 patients, which could indicate that gum chewing may be a reversible risk factor for some individuals with TMD. Yashiro *et al*. [41] identified that patients with TMD had higher average UV during gum-chewing compared to controls. This suggests that individuals with TMD exhibit greater abnormalities in chewing movements, which may be indicative of underlying motor control dysfunction.

## 4. Discussion

Upon examination of the collective findings of the studies they collectively offer a spectrum of findings where some [34, 35, 37] lean towards a minimal or non-causal relationship between gum chewing and TMD pain, while others [36] indicate a more contributory role of extensive gum chewing in TMD symptomatology. The remaining studies [38, 39, 40, 41] provide additional depth by exploring the neuromuscular and biomechanical aspects of TMD, illustrating the multifaceted nature of the condition and the various methodologies used to investigate it. Al Sayegh *et al*. [34] and Christensen *et al*. [35] both reported findings that question the direct association between gum chewing and the development of TMD-related pain. Al Sayegh *et al*. [34] suggested that symptoms decreased after cessation of the task and varied with chewing duration, which could imply that the relationship between gum chewing and TMD is not causal or may be influenced by other mediating factors. Similarly, Christensen *et al*. [35] found no evidence of myofascial pain, inferring that muscle fatigue due to gum chewing does not equate to TMD-related pain. Therefore, both studies cast doubt on the sufficiency of gum chewing as a sole etiological factor for TMD pain.

Conversely, Correia *et al*. [36] offered a dissimilar perspective, associating high frequency and longer duration of gum chewing with an increased incidence of TMD symptoms and masseter hypertrophy. This observation indicates a potential dose-response relationship between the extent of gum chewing and the exacerbation of TMD symptoms, a point of divergence from the conclusions drawn by Al Sayegh *et al*. [34] and Christensen *et al*. [35]. Farella *et al*. [37] contributed to the discourse by suggesting that jaw muscles, particularly in non-TMD individuals, show a robust capacity for recovery from the stress of prolonged chewing, indicating a resilience that may protect against the development of TMD. This finding is similar to that of Christensen *et al*. [35] in that it does not support a strong link between gum chewing and TMD pain, but it diverges from Correia *et al*. [36] by suggesting that any discomfort from gum chewing is transitory and non-pathological in individuals without pre-existing TMD.

The work of Matsuda *et al*. [38] introduced a distinct aspect of TMD-related muscle activity, illustrating that RMMA patterns during sleep differ significantly from those during gum chewing. This study diverges from all previously mentioned studies [34, 35, 36, 37] as it does not directly assess the impact of gum chewing on TMD symptoms but instead provides insight into the neuromuscular discrepancies between pathological and non-pathological masticatory muscle activity. Olchowy *et al*. [39] further diversified the range of findings by introducing the reversibility of muscle stiffness following intense gum chewing, adding a biomechanical dimension to the understanding of TMD. This study, while somewhat aligned with Farella *et al*. [37] regarding the reversibility of muscle strain, differs from Correia *et al*. [36] which suggested more persistent changes in muscle structure.

Watemberg *et al*. [40] found no correlation between the volume of daily gum chewing and the improvement of headache symptoms upon cessation, a result that diverges from Correia *et al*. [36] by implying that other factors may be more influential in TMD-related headaches than mechanical stress from gum chewing. Yashiro *et al*. [41] provided a methodological perspective, utilizing the minimum jerk model to analyze gum-chewing kinematics. The finding that the model predicted movements accurately in healthy adults but not in TMD patients is dissimilar to the other studies, as it focuses on the assessment of movement patterns rather than direct symptomatology or muscle response.

The conceptualization of stomatognathic adaptive motor syndrome as a diagnostic category for the complexities of TMDs was advanced by Douglas *et al*. [42]. This theoretical framework posits that suboptimal dental occlusion and mandibular alignment necessitate compensatory mandibular micro-movements, which may incite a cascade of adaptive responses within the stomatognathic architecture. Hallmarks of this syndrome encapsulate the conventional symptomatology of TMD. Within the domain of masticatory musculature, clinical manifestations may include myalgia, hypertonicity, fatigue and diminished strength, with muscle hypertonicity quantifiable through shear wave elastography, evidenced by an escalation in muscular stiffness [42].

Patients with this syndrome frequently report challenges in masticating firmer foodstuffs, attributable to the triad of pain, fatigue and reduced muscular power. Dietary modifications towards softer food intake have been posited to attenuate the reactive sequelae in temporomandibular joint tissue, potentially alleviating symptomatology [42]. The interrelationship between dietary practices, parafunctional behaviors such as habitual gum chewing, and the heightened risk of TMD beckons require further exploration to enhance diagnostic precision and therapeutic outcomes. Investigations into the array of factors influencing masticatory efficacy could yield actionable insights for the adjunctive management of TMD.

The proposition that augmenting masticatory muscle fortitude may offer therapeutic advantages for TMD patients finds support in the literature. Notably, one paper [43] documented more pronounced pain alleviation and a significant decrement in disability scores following a regimen of controlled masticatory exercises. These exercises were notably contrasted with the act of masticating more resistant substances, such as wax, emphasizing the role of exercise intensity in therapeutic outcomes. Complementarily, Kim* et al*. [44] observed in an older cohort that gum chewing may confer a suite of benefits, including enhanced bite force, salivation and improved deglutition.

Shear wave elastography emerges as a promising modality for assessing masseter muscle stiffness [45]. Investigations have revealed that intensive gum chewing markedly elevates stiffness within the masticatory musculature, a condition that persists beyond the cessation of the activity. Comparative analysis indicated that the masseter muscle consistently exhibited higher stiffness indices at assorted measurement intervals. Beyond physical exertion, other factors have been identified as influencers of muscle stiffness. For instance, a reduction in masseter muscle stiffness post-massage was documented by Olchowy C *et al*. [46], who observed a statistically significant decrease in stiffness metrics in their cohort. Additionally, Ariji *et al*. [47] employed sonographic elastography to evaluate masseter muscle stiffness in TMD patients with myofascial pain, reporting a significant reduction in median elasticity index ratios among the responder group after treatment sessions, while the non-responder group’s reduction in stiffness did not achieve statistical significance.

The findings from Lippi *et al*. [9] and Jabr *et al*. [48], while focusing on different conditions, share a common theme in exploring the relationship between gum-chewing and pain, albeit in distinct contexts. Lippi *et al*. [9] reviewed literature regarding the potential connection between gum-chewing and headache, particularly in individuals with migraine or tension-type headache. Their conclusion suggested a possible trigger effect of gum-chewing in such individuals, advising caution in this activity for those suffering from these types of headaches. In contrast, non-migraineurs did not experience an increase in headache prevalence after gum-chewing, which points to a specific vulnerability in migraineurs and tension-type headache patients to gum-chewing as a pain trigger. Jabr *et al*. [48], on the other hand, investigated the effects of chewing sugar-free gum on self-reported orthodontic treatment pain, comparing it with conventional analgesic drugs (CADs). Their methodology involved a meta-analysis of RCTs, which is a systematic approach similar to the review process Lippi *et al*. [9] used. However, the focus of Jabr *et al*. [48] was narrower, examining the efficacy of gum-chewing in pain alleviation during orthodontic treatment. The results from the meta-analysis indicated no significant difference in pain scores between the sugar-free gum group and the ibuprofen group at various time points following orthodontic appliance placement. This finding suggests that gum-chewing did not exacerbate pain and was comparable to ibuprofen in terms of pain management in the context of orthodontic pain.

Comparing these findings to our study, both Lippi *et al*. [9] and Jabr *et al*. [48] present the idea that gum-chewing does not universally cause or exacerbate pain but may have situational effects depending on the population and the type of pain being investigated. In terms of similarities, our study, along with Lippi *et al*. [9], identifies a subset of individuals (TMD sufferers) who may experience symptom exacerbation due to gum-chewing, akin to migraineurs and tension-type headache sufferers. However, our findings also align with Jabr *et al*. [48] in that gum-chewing does not necessarily lead to an increase in pain symptoms for everyone, and in some cases, it may not significantly differ from other forms of treatment such as CADs in managing pain.

### 4.1 Limitations

The investigations into the association between gum chewing and TMDs encountered several limitations that warrant consideration when interpreting the findings. One prominent limitation was the inherent heterogeneity among study designs, including variations in sample size, population characteristics, and methodological approaches. This diversity among studies introduced challenges in synthesizing data and generating a cohesive understanding of the relationship between gum chewing and TMDs. Furthermore, the duration of the studies was generally short-term, which may not accurately capture the long-term effects of gum chewing on the temporomandibular joint and associated musculature. Consequently, the potential for reverse causation or the presence of confounding factors that might influence the onset or progression of TMD symptoms was not thoroughly examined. This is particularly relevant for observational studies where the control for external variables is inherently limited, and causality cannot be definitively established. In addition, the studies predominantly focused on the mechanical aspects of gum chewing, with less attention given to the biochemical or molecular mechanisms that may contribute to TMD pathogenesis. This omission suggests that the studies may not have captured the full spectrum of factors that influence TMDs, which could be critical to understanding the disorder’s multifactorial nature.

### 4.2 Clinical recommendations and implications for future research

Our results imply numerous suggestions depending on the material given. First of all, extended chewing activities—such as gum chewing—should be done carefully since they could aggravate TMJ problems and muscle tiredness. To reduce possible detrimental consequences, people should thus be aware of the length of their chewing activities. Also, considering the consequences of gum chewing, one must choose a customised method. Gum chewing can have different effects on different people; some may get musculoskeletal changes, pain syndromes, or muscle hypertrophy. Consequently, advice on gum chewing should be customised to the particular demand and situation of every person.

Furthermore, advised is consistent observation of symptoms and their reaction to gum chewing or other masticatory action. This enables people to spot any changes in the degree or length of symptoms after chewing activities, therefore enabling the identification of possible triggers and the application of suitable treatment techniques. Moreover, it is important to admit that extended chewing activities could cause muscle tiredness. People should be conscious of their degree of muscle tiredness and provide top priority to enough rest and recovery so that the chewing-related muscles could heal.

Future research should standardize methodologies to assess the impact of gum chewing on TMD, including consistent chewing durations and types. Longitudinal studies are needed to examine the long-term effects of habitual gum chewing on TMD development and recovery periods. Specific symptoms like arthralgia, myofascial pain and muscle fatigue should be differentiated to understand their underlying mechanisms. Research should also explore the interaction between psychological factors and physical symptoms. Detailed investigations into muscle activity, stiffness, and kinematics during chewing tasks can provide insights into the biomechanical and neuromuscular aspects of TMD. The potential reversibility of symptoms with the cessation of gum chewing and identifying susceptible subgroups could inform preventive and therapeutic strategies.

## 5. Conclusions

Investigations included in this review that were undertaken demonstrated a spectrum of conclusions, with a segment of studies suggesting a lack of a direct causal linkage between gum chewing and the manifestation of TMD-related pain. These studies posited that any observed symptoms were transient and often abated following cessation of the chewing activity, which implies that factors other than the mechanical act of chewing may play a more salient role in the pathogenesis of TMD. In contrast, another subset of research identified a more pronounced relationship, with evidence indicating that the frequency and duration of gum chewing correlated with an uptick in TMD symptoms, including muscle discomfort and hypertrophy. This relationship hints at a possible dose-dependent effect, where the intensity of chewing is directly proportional to symptom severity. Furthermore, some studies in this domain emphasized the resiliency of jaw musculature, especially in individuals without pre-existing TMD, suggesting that muscle recovery post-chewing may serve as a protective mechanism against the development of TMD. Additional dimensions to the conversation emerged from studies focusing on neuromuscular and biomechanical responses to gum chewing. These studies highlighted the distinct electromyographic patterns associated with TMD as opposed to normal muscle activity, underscoring the potential for certain diagnostic tools to differentiate between normal and pathological masticatory function. Observations were made noting the reversible nature of muscle stiffness resulting from intense gum chewing, which broadens the understanding of the biomechanical responses within the masticatory musculature.

## Supplementary Material

Supplementary material associated with this article can be found, in the online version, at https://files.jofph.com/files/article/1904342541406224384/attachment/Supplementary%20material.docx.


